# Keeping it simple – a Simple Behavioral Analysis (SimBA) primer

**DOI:** 10.1038/s44277-024-00014-9

**Published:** 2024-08-02

**Authors:** Nastacia L. Goodwin, Sam A. Golden

**Affiliations:** 1 University of Washington, Department of Biological Structure, Seattle, WA USA; 2 University of Washington, Graduate Program in Neuroscience, Seattle, WA USA; 3 University of Washington, Center of Excellence in Neurobiology of Addiction, Pain, and Emotion (NAPE), Seattle, WA USA

**Keywords:** Neuroscience, Computational neuroscience

Neuroscience is approaching a critical juncture, transitioning from subjective behavioral observation to objective behavioral definition. This is now possible due to analytical tools using machine-guided solutions for animal markerless key-point pose tracking (see Table 1 for specialized terminology definitions) and behavioral detection. However, it is often challenging for behaviorists to adopt these approaches due to a lack of specialized computational knowledge. We and others have spent significant effort to solve this problem by democratizing access to these approaches, making them accessible to non-specialists. However, as these tools increase access to machine learning approaches, an emerging problem is ensuring that the predictions algorithms are making and the biology that they are defining are explainable and understandable. To address these challenges, we have introduced Simple Behavioral Analysis (SimBA) [1] as an open-source, supervised machine learning based platform for the automated detection of behavior, with a focus on understanding how the underlying machine learning algorithms make their decisions.

**Table 1:** Glossary of terms commonly used in machine learning.

Term or acronym	Definition
Markerless pose tracking	Tracking of unmarked animal body parts in video frames, such as ears and paws. Commonly performed by open-source programs such as SLEAP and DLC.
Key-point	Location of animal body part of interest obtained from markerless pose tracking.
Feature	Calculation about the relationship between key-points (i.e. distance between noses).
Classifier	Algorithm that tells you the probability of a behavior of interest occurring in a given video frame.
Explainability	The ability to understand why an algorithm gives you particular results based on your input data.
Closed-loop	Stimulation or other manipulations are automatically triggered by near real-time prediction or detection of behavior.
Supervised algorithm	An algorithm for which you provide a dataset of positive and negative examples of behaviors so that it can learn the difference between what you want to detect and what it should disregard. SimBA uses binary supervised classifiers, such as “attack” or “not attack” for each behavior. Supervised classifiers are generally considered easier to use than unsupervised classifiers.
Unsupervised algorithm	An algorithm for which you provide sets of rules regarding mathematical similarities and differences so that it may parse datasets into behavioral clusters that it finds independently. Unsupervised algorithms are powerful in that they may find novel behavioral motifs, but they can be difficult to tune and interpret.
SimBA	Simple Behavioral Analysis – An open-source program which takes in pose-estimation and outputs an explainable probability of a behavior of interest.
SHAP	Shapley Additive Explanations – A game theory-based approach to machine learning explainability.

**Fig. 1:**
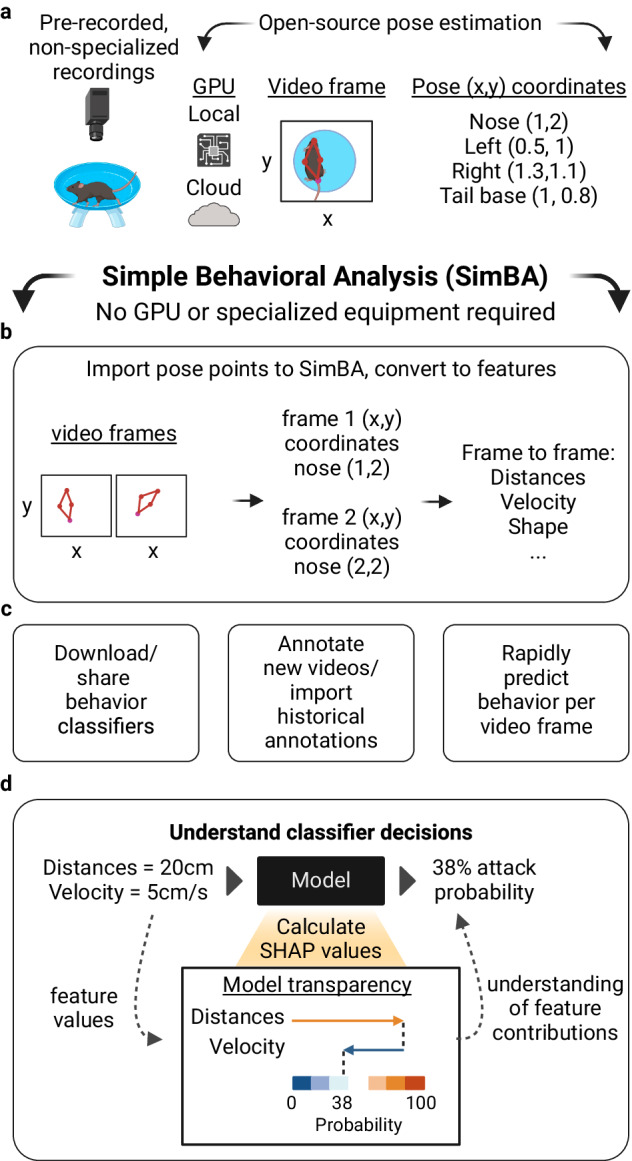
SimBA workflow. **a** SimBA accepts pre-recorded and non-specialized video recordings, which can then be processed for pose-estimation through open-source programs such as SLEAP or DeepLabCut. **b** Pose data are imported into SimBA, which calculates hundreds of features per video frame. Features are then used to predict behaviors on a frame-by-frame basis. **c** Users can download and share behavioral classifiers, can import historical annotations, or can annotate new videos in SimBA. **d** Users can then use SHAP explainability scores to understand why their classifiers are giving them particular results, and the behavioral nuance in their experimental groups.

SimBA extends the use of markerless key-point pose tracking provided by open-source programs such as SLEAP [2] and DeepLabCut [3] – machine vision programs that learn to detect animal body parts (key-points) in the video – transforming tracking data into relationships between body parts over different durations (‘features’, i.e. distance between animal noses). Researchers can then use SimBA to train a supervised algorithm, called a classifier, to detect behaviors of interest based on these feature values. Importantly, SimBA provides easy access to explainable artificial intelligence tools such as Shapley Additive exPlanations [4] (SHAP) to understand why specific feature values lead to particular predictions (Fig. 1). For example, in a simple social interaction where two animals are socially exploring each other, there are many features that might define that interaction. However, features related to proximity and velocity may better define this behavioral state than features related to body shape or size. SHAP values provide a quantitative value for every feature used in predicting whether that social interaction is occurring. These explainability values define these behavioral states. SHAP is a widely cited open-source and post-hoc explainability method that can be applied and compared across any of the current machine learning platforms regardless of the pose estimation scheme.

By computing and sharing these explainability metrics, we can reconceptualize behavioral classifiers as objective and shareable reagents akin to the commonly used Research Reagent Identifiers (RRIDs) system for wet lab reagents [5], rather than relying on a few sentences in the methods to try to standardize behavioral definitions (i.e., attacks consisted of bites and lunges). This objective quantification of behavioral classifiers is a key advancement toward complying with the National Institutes of Health rigor and reproducibility push.

**Key features**

- Users can create and share classifiers across labs to standardize behavioral definitions.
- Users can analyze archived video datasets and newly acquired data, directly.
- In-line access to SHAP calculations allows users to objectively quantify and report their behaviors of interest.
- Automated behavioral analysis provides a frame-by-frame probability of behaviors of interest occurring in a rapid and reproducible manner.

**High-impact applications**

- High throughput behavioral screening in drug development and therapeutics, such as tracking social interactions and locomotor activity following drug administration.
- SimBA has been used for classical ethological analysis in model organisms including mice, rats, fish, wasps, moths, and birds.
- Detected behaviors can then be mathematically clustered into distinct behavioral motifs using unsupervised learning techniques.
- Accessible explainability metrics allow for quantitative, rather than qualitative, definitions of complex behavioral states.

**Limitations and aspirational refinements**

- Supervised machine learning is limited to detecting previously defined behaviors.
- Classifier creation in SimBA does require a small amount of hand annotation of behaviors.
- We hope to extend SimBA to real time closed loop applications, in which an algorithm can predict or detect a behavior in real-time and trigger a manipulation, such as optogenetic stimulation, in the future.
